# Supplementary material for: Changes in PRC1 activity during interphase modulate lineage transition in pluripotent cells
Source: Nat Commun. 2023 Jan 12;14:180. doi: 10.1038/s41467-023-35859-9 (PMC9837203; doi:10.1038/s41467-023-35859-9)
Supplement: Supplementary file 1 — Supplementary Information [file 41467_2023_35859_MOESM1_ESM.pdf]

Supplementary Information

Changes in PRC1 activity during interphase modulate lineage transition in pluripotent cells

Helena G. Asenjo[1,2,3], María Alcazar-Fabra[1,2,3], Mencía Espinosa-Martínez[1,2,3], Lourdes Lopez-Onieva[1,4], Amador Gallardo[1,2,3], Emilia Dimitrova[5], Angelika Feldmann[5,10], Tomas Pachano[6], Jordi Martorell-Marugán[1, 7, 8], Pedro Carmona-Sáez[1,7,9], Antonio Sanchez-Pozo[1,2,3], Álvaro Rada-Iglesias[6], Robert J. Klose[5], David Landeira[1,2,3]

[1]Centre for Genomics and Oncological Research (GENYO), Avenue de la Ilustración 114, 18016 Granada, Spain.

[2]Department of Biochemistry and Molecular Biology II, Faculty of Pharmacy, University of Granada, Granada, Spain.

[3]Instituto de Investigación Biosanitaria ibs.GRANADA, Granada, Spain.

[4]Department of Biochemistry and Molecular Biology I, Faculty of Sciences, University of Granada, Granada, Spain.

[5]Department of Biochemistry, University of Oxford, Oxford, UK.

[6]Institute of Biomedicine and Biotechnology of Cantabria (IBBTEC), CSIC/Universidad de Cantabria, Santander, Spain.

[7]Department of Statistics and Operational Research, University of Granada, Granada 18071, Spain.

[8]Data Science for Health Research Unit, Fondazione Bruno Kessler, Trento 38123, Italy.

[9]Excellence Research Unit "Modelling Nature", University of Granada, Granada, Spain.

[10]Current affiliation: German Cancer Research Center (DKFZ), Heidelberg, Germany.

Correspondence to David Landeira: davidlandeira@ugr.es

Running title: Polycomb favours cell differentiation during the G1 phase

Keywords: Epigenetics, chromatin, polycomb repressive complex, PRC1, mESCS, pluripotency, cell cycle, cell differentiation.

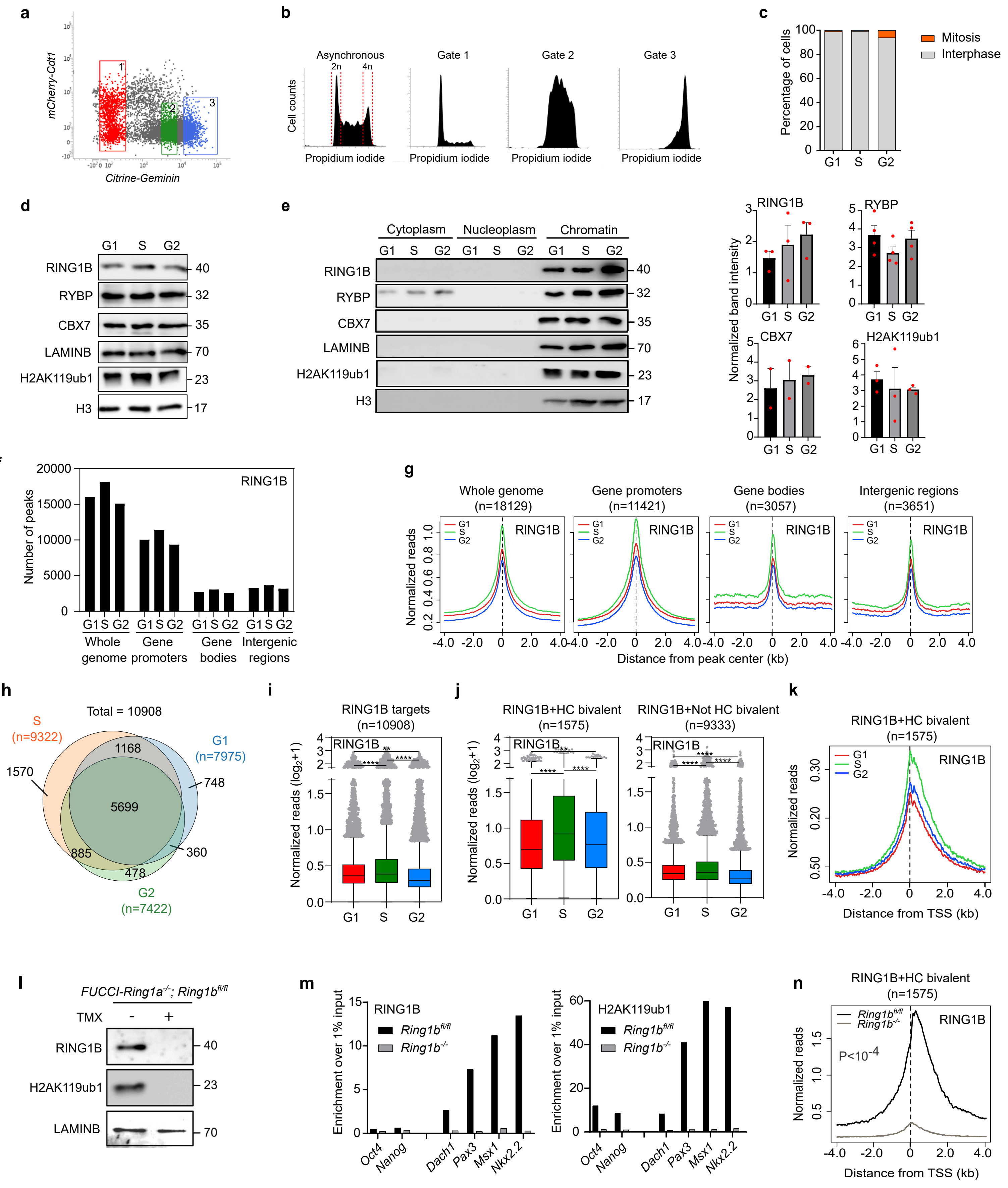
a
mCherry-Cdt1
Citrine-Geminin
b
Asynchronous
Gate 1
Gate 2
Gate 3
2n
4n
Cell counts
Propidium iodide
c
Percentage of cells
Mitosis
Interphase
G1 S G2
d
G1 S G2
RING1B
RYBP
CBX7
LAMINB
H2AK119ub1
H3
e
Cytoplasm
Nucleoplasm
Chromatin
Normalized band intensity
f
Number of peaks
RING1B
Whole genome
Gene promoters
Gene bodies
Intergenic regions
g
Whole genome (n=18129)
Gene promoters (n=11421)
Gene bodies (n=3057)
Intergenic regions (n=3651)
Normalized reads
Distance from peak center (kb)
h
Total = 10908
S (n=9322)
G1 (n=7975)
G2 (n=7422)
1168
1570
748
5699
885
360
478
i
RING1B targets (n=10908)
Normalized reads (log2+1)
j
RING1B+HC bivalent (n=1575)
RING1B+Not HC bivalent (n=9333)
k
RING1B+HC bivalent (n=1575)
Distance from TSS (kb)
l
FUCCI-Ring1a-/-; Ring1bfl/fl
TMX
m
Enrichment over 1% input
Ring1bfl/fl
Ring1b-/-
Oct4
Nanog
Dach1
Pax3
Msx1
Nkx2.2
n
RING1B+HC bivalent (n=1575)
P<10-4
Distance from TSS (kb)

Supplementary Figure 1. The global amount of PRC1 subunits on chromatin is similar during G1, S and G2 phases.

(a) Flow cytometry dot plot analysis of wild-type FUCCI-mESCs (expressing mCherry-Cdt1 and Citrine-Geminin fusion proteins) indicating the sorting gates used to obtain cell populations enriched in G1 (Gate 1), S (Gate 2) and G2 (Gate 3) phases of the cell cycle.

(b) Flow cytometry analysis of DNA content in cells stained with propidium iodide after sorting as indicated in (a).

(c) Percentage of cells in interphase (grey) or mitosis (orange) at indicated fractions after cell cycle sorting as assayed by fixation, DAPI staining and microscopy analysis. Percentage of mitotic cells in G1, S and G2 fractions were 0.98%, 0.7% and 6.3% respectively.

(d) Whole cell lysate western blots of wild-type FUCCI-mESCs comparing the level of RING1B, RYBP, CBX7 and H2AK119ub1 in G1, S and G2 phases. Lamin B and histone 3 (H3) were used as a loading control. Molecular weight is indicated in kDa.

(e) Western blot analyses of RING1B, RYBP, CBX7 and H2AK119ub1 proteins upon biochemical cell fractionation of wild-type FUCCI-mESCs in the indicated cell-cycle phases. Lamin B and histone 3 (H3) were used as loading control for the chromatin fraction. Molecular weight is indicated in kDa. Histograms show quantification of the intensity of the bands in the chromatin fraction (using Lamin B and H3 as normalizers). Mean ± SEM of three independent experiments showed no significant difference using Mann-Whitney test.

(f) Histogram showing the number of peaks detected in the RING1B ChIP-seq across the whole genome, gene promoters (±2 kb from TSS), gene bodies and intergenic regions at indicated cell cycle phases.

(g) Average signal of RING1B binding at peaks detected in S phase and categorized as in (f) in G1 (red), S (green) and G2 (blue) phases.

(h) Venn diagram showing the overlap between RING1B target promoters detected in G1, S and G2 phases. Total number of promoters bound by RING1B at any phase of the cell cycle is indicated (n=10908).

(i, j) Quantification of RING1B-binding signal at the promoter region (-0.5 kb to +1.5 kb relative to TSS) of target genes (i) that are HC bivalent or not (j) (as identified in figure 1D) at the indicated cell cycle phases. Boxes show median and Q1-Q3 values. Whiskers denote the 1.5X the interquartile range. Mann-Whitney test was applied (*P<0.05, **P<0.01, ***P<0.001, ****P<0.0001).

(k) Average binding profile of RING1B around the TSS of HC bivalent RING1B target promoters in G1 (red), S (green) and G2 (blue) phases using an alternative anti-RING1B antibody (MBL D139-3) for ChIP-seq.

(l) Western blots of whole cell extracts of RING1B and H2AK119ub1 in FUCCI-*Ring1a*$^{-/-}$;*Ring1b*$^{fl/fl}$ mESC before (-) and after (+) 72h Tmx treatment. Lamin B was used as loading control. Molecular weight is indicated in kDa.

(m) Analysis by ChIP-qPCR comparing the enrichment of RING1B (left) and H2AK119ub1 (right) at candidate PRC1-target promoter regions (Dach1, Pax3, Msx1, Nkx2-2) in an asynchronous population of parental FUCCI-*Ring1a*$^{-/-}$;*Ring1b*$^{fl/fll}$ and 72h Tmx-treated *Ring1b*$^{-/-}$. Active (Oct4, Nanog) gene promoters were used as negative controls.

(n) Average binding signal of RING1B around the TSS of HC bivalent target promoters in parental FUCCI-*Ring1a*$^{-/-}$;*Ring1b*$^{fl/fl}$ (black line) and 72h Tmx-treated *Ring1b*$^{-/-}$ (grey line) mESCs. *P* indicates nonparametric Mann-Whitney test p-value.

Source data are provided as a source data file.

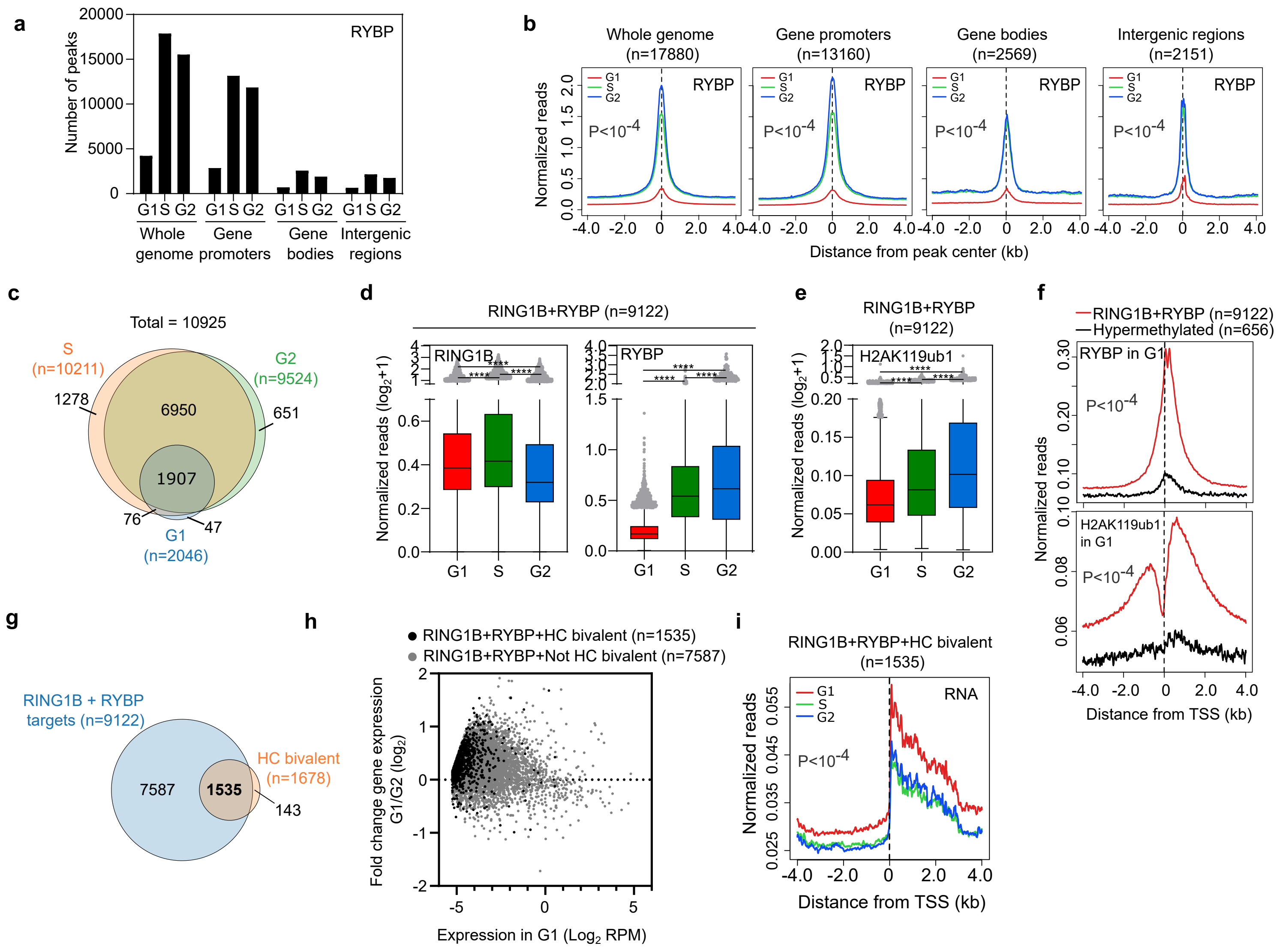

a
RYBP
Number of peaks
20000
15000
10000
5000
0
G1 S G2
Whole genome
Gene promoters
Gene bodies
Intergenic regions
b
Whole genome (n=17880)
Gene promoters (n=13160)
Gene bodies (n=2569)
Intergenic regions (n=2151)
Normalized reads
P<10-4
Distance from peak center (kb)
c
Total = 10925
S (n=10211)
G2 (n=9524)
G1 (n=2046)
1278
6950
651
1907
76
47
d
RING1B+RYBP (n=9122)
RING1B
RYBP
Normalized reads (log2+1)
e
RING1B+RYBP (n=9122)
H2AK119ub1
f
RING1B+RYBP (n=9122)
Hypermethylated (n=656)
RYBP in G1
H2AK119ub1 in G1
Distance from TSS (kb)
g
RING1B + RYBP targets (n=9122)
HC bivalent (n=1678)
7587
1535
143
h
RING1B+RYBP+HC bivalent (n=1535)
RING1B+RYBP+Not HC bivalent (n=7587)
Fold change gene expression G1/G2 (log2)
Expression in G1 (Log2 RPM)
i
RING1B+RYBP+HC bivalent (n=1535)
RNA
Distance from TSS (kb)

Supplementary Figure 2. Rybp and H2AK119ub1 are recruited to target promoters during G1 phase in mESCs.

(a) Histogram showing the number of peaks detected in the RYBP ChIP-seq across the whole genome, gene promoters (±2 kb from TSS), gene bodies and intergenic regions at indicated cell cycle phases.

(b) Average signal of RYBP binding at peaks detected in S phase and categorized as in (f) in G1 (red), S (green) and G2 (blue) phases. *P* indicates one-way ANOVA test p-value.

(c) Venn diagram showing the overlap between RYBP target promoters detected in G1, S and G2 phases. Total number of promoters bound by RYBP at any phase of the cell cycle is indicated (n=10925).

(d, e) Quantification of RING1B, RYBP (d) and H2AK119ub1 (e) binding signal at the promoter region (-0.5 kb to +1.5 kb relative to TSS) of RING1B and RYBP targets in the indicated phases of the cell cycle. Boxes show median and Q1-Q3 values. Whiskers denote the 1.5X the interquartile range. Mann-Whitney test was applied (*P<0.05, **P<0.01, ***P<0.001, ****P<0.0001).

(f) Average enrichment profile of RYBP (top) and H2AK119ub1 (bottom) around the TSS at RING1B and RYBP target promoters (red) compared to hypermethylated promoters (black) in G1 phase. Hypermethylated promoters were previously defined [1]. *P* indicates nonparametric Mann-Whitney test p-value.

(g) Venn diagram showing the overlap between HC bivalent promoters (orange) and RING1B+RYBP target promoters (red).

(h) MA plot showing fold change gene expression between cells in G1 and G2 phases (4sU-seq normalized reads mapping from the TSS to +3kb) at HC bivalent RING1B+RYBP targets (black dots) and Not HC bivalent RING1B+RYBP targets (grey dots). Nascent RNA expression in G1 is represented in the x-axis.

(i) Average 4sU-seq RNA reads at RING1B+RYBP+HC bivalent promoters in G1 (red), S (green) and G2 (blue) phases. *P* indicates one-way ANOVA test p-value.

Source data are provided as a source data file.

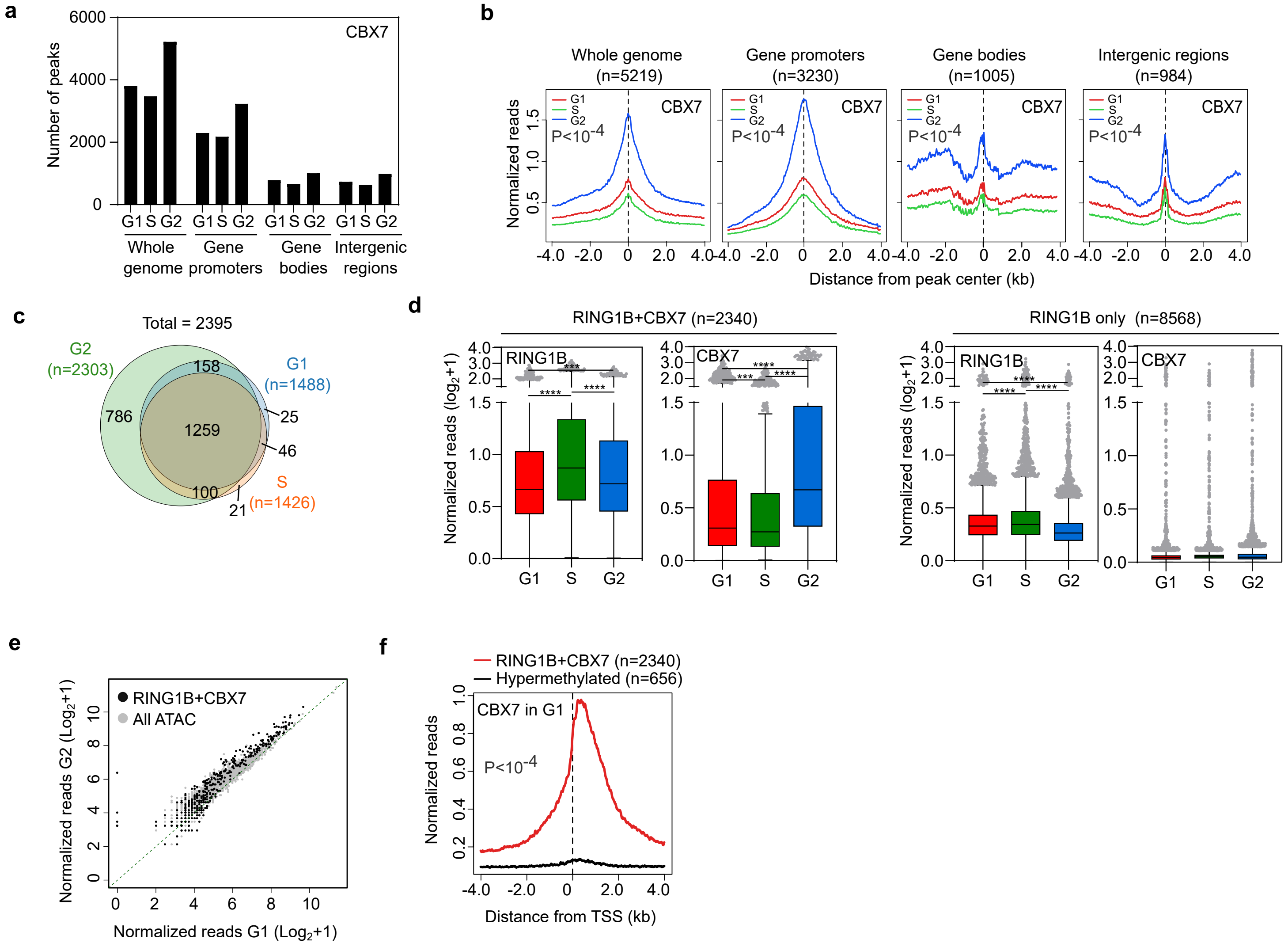
a
CBX7
Number of peaks
6000
4000
2000
0
G1 S G2
Whole genome
Gene promoters
Gene bodies
Intergenic regions
b
Whole genome (n=5219)
Gene promoters (n=3230)
Gene bodies (n=1005)
Intergenic regions (n=984)
Normalized reads
P<10-4
Distance from peak center (kb)
c
Total = 2395
G2 (n=2303)
G1 (n=1488)
S (n=1426)
158
786
1259
25
46
100
21
d
RING1B+CBX7 (n=2340)
RING1B only (n=8568)
RING1B
CBX7
Normalized reads (log2+1)
e
RING1B+CBX7
All ATAC
Normalized reads G2 (Log2+1)
Normalized reads G1 (Log2+1)
f
RING1B+CBX7 (n=2340)
Hypermethylated (n=656)
CBX7 in G1
P<10-4
Normalized reads
Distance from TSS (kb)

Supplementary Figure 3. Cbx7 is recruited to target promoters during G1 phase in mESCs.

(a) Histogram showing the number of peaks detected in the CBX7 ChIP-seq across the whole genome, gene promoters (±2 kb from TSS), gene bodies and intergenic regions at indicated cell cycle phases.

(b) Average signal of CBX7 binding at peaks detected in S phase and categorized as in (f) in G1 (red), S (green) and G2 (blue) phases. *P* indicates one-way ANOVA test p-value.

(c) Venn diagram showing the overlap between CBX7 target promoters detected in G1, S and G2 phases. Total number of promoters bound by CBX7 at any phase of the cell cycle is indicated (n=2395).

(d) Quantification of RING1B and CBX7 binding signal at the promoter region (-0.5 kb to +1.5 kb relative to TSS) of RING1B-bound promoters that are targeted by CBX7 (left panel) or not (right panel) at the indicated phases of the cell cycle. Boxes show median and Q1-Q3 values. Whiskers denote the 1.5X the interquartile range. Mann-Whitney test was applied (*P<0.05, **P<0.01, ***P<0.001, ****P<0.0001).

(e) Scatter plot showing the Capture-C signal in G1 (x axis) compared to G2 (y axis) of interactions (n=2034) taking place between accessible promoters (n=179) and other regions of the genome (black and grey dots). Black dots highlight interactions (n=392) involving RING1B+CBX7-bound promoters (n=39).

(f) Average enrichment profile of CBX7 around the TSS at RING1B+CBX7 target promoters (red) compared to hypermethylated promoters (black) in G1 phase. Hypermethylated promoters were previously defined [1]. *P* indicates nonparametric Mann-Whitney test p-value.

Source data are provided as a source data file.

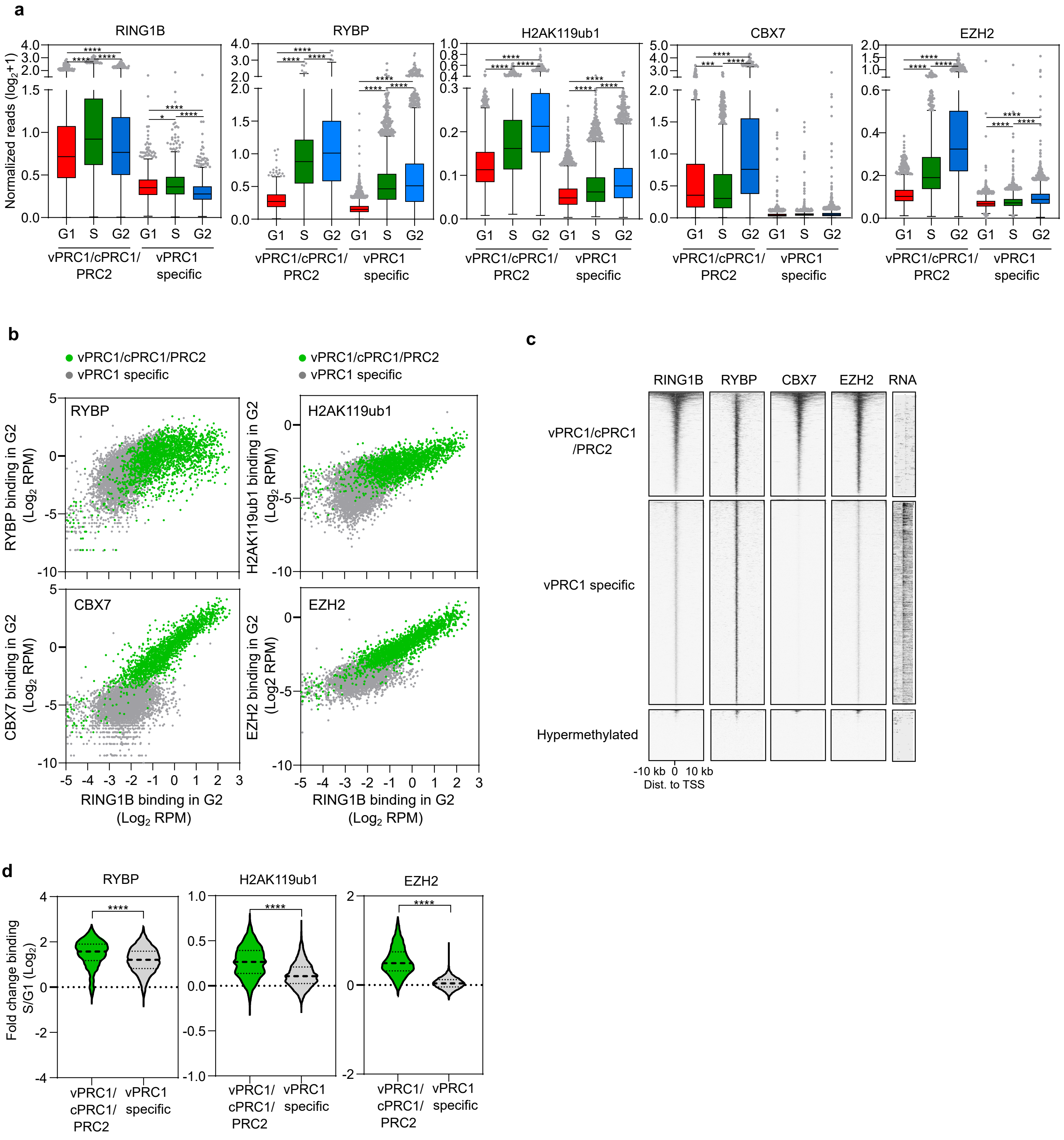
a
RING1B
RYBP
H2AK119ub1
CBX7
EZH2
Normalized reads (log2+1)
G1 S G2 G1 S G2
vPRC1/cPRC1/ PRC2
vPRC1 specific
b
vPRC1/cPRC1/PRC2
vPRC1 specific
RYBP binding in G2 (Log2 RPM)
H2AK119ub1 binding in G2 (Log2 RPM)
CBX7 binding in G2 (Log2 RPM)
EZH2 binding in G2 (Log2 RPM)
RING1B binding in G2 (Log2 RPM)
c
RING1B
RYBP
CBX7
EZH2
RNA
vPRC1/cPRC1 /PRC2
vPRC1 specific
Hypermethylated
-10 kb 0 10 kb
Dist. to TSS
d
RYBP
H2AK119ub1
EZH2
Fold change binding S/G1 (Log2)
vPRC1/ cPRC1/ PRC2
vPRC1 specific

Supplementary Figure 4. vPRC1/cPRC1/PRC2 target genes display higher levels of Ring1b, Rybp, Cbx7, Ezh2 and H2AK119ub1 binding than vPRC1 specific target genes.

(a) Quantification of RING1B, RYBP, H2AK119ub1, CBX7 and EZH2 binding signal at the promoter region (-0.5 kb to +1.5 kb relative to TSS) of vPRC1/cPRC1/PRC2 (n=2093) and vPRC1 specific target promoters (n=6222) in the indicated cell cycle phases. Boxes show median and Q1-Q3 values. Whiskers denote the 1.5X the interquartile range. Mann-Whitney test was applied (*P<0.05, **P<0.01, ***P<0.001, ****P<0.0001).

(b) Correlation analysis between the binding signals (-0.5 kb to +1.5 kb relative to TSS) of RYBP, H2AK119ub1, CBX7 or EZH2 compared to RING1B in G2 phase at vPRC1/cPRC1/PRC2 (green dots) and vPRC1 specific (grey dots) target promoters.

(c) Heatmaps of normalized RING1B, RYBP, CBX7 and EZH2 ChIP-seq reads and RNA expression around the TSS (±10 kb) of vPRC1/cPRC1/PRC2, vPRC1 specific and control hypermethylated promoters in G2 phase. Genes are ranked according to the signal of RING1B binding.

(d) Violin plots showing the fold change binding between S and G1 of RYBP, H2AK119ub1 and EZH2 at promoter regions (-0.5 kb to +1.5 kb relative to TSS) of vPRC1/cPRC1/PRC2 (green) and vPRC1 specific target genes (grey). P values were calculated using Mann-Whitney test. Asterisks (****) mark statistically significant differences (P<0.0001).

Source data are provided as a source data file.

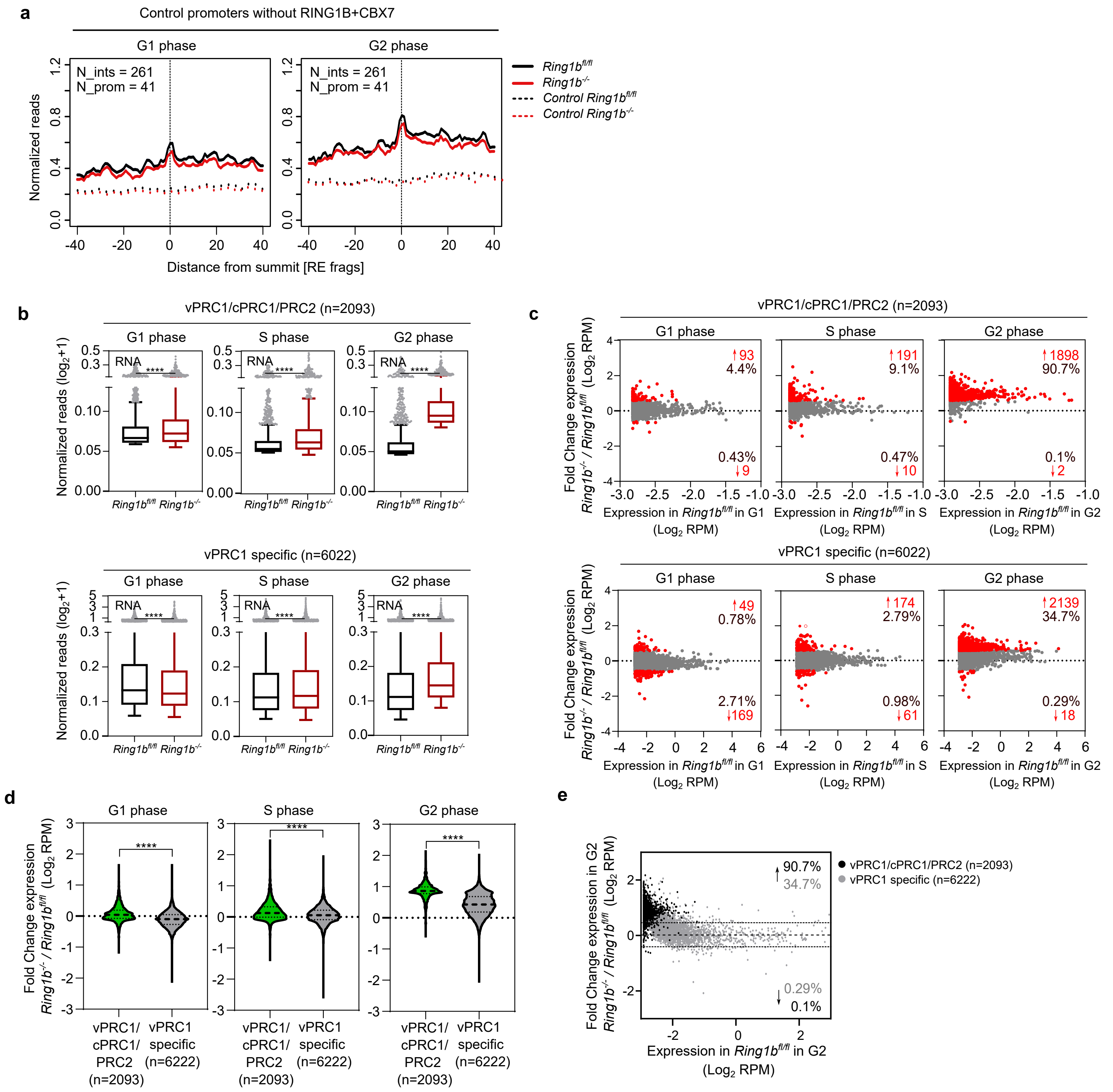
a
Control promoters without RING1B+CBX7
G1 phase
G2 phase
N_ints = 261
N_prom = 41
Normalized reads
Distance from summit [RE frags]
Ring1b fl/fl
Ring1b -/-
Control Ring1b fl/fl
Control Ring1b -/-
b
vPRC1/cPRC1/PRC2 (n=2093)
G1 phase
S phase
G2 phase
RNA
****
Normalized reads (log2+1)
vPRC1 specific (n=6022)
c
vPRC1/cPRC1/PRC2 (n=2093)
Fold Change expression Ring1b-/- / Ring1b fl/fl (Log2 RPM)
93
4.4%
0.43%
9
191
9.1%
0.47%
10
1898
90.7%
0.1%
2
Expression in Ring1b fl/fl in G1 (Log2 RPM)
Expression in Ring1b fl/fl in S (Log2 RPM)
Expression in Ring1b fl/fl in G2 (Log2 RPM)
vPRC1 specific (n=6022)
149
0.78%
2.71%
169
174
2.79%
0.98%
61
2139
34.7%
0.29%
18
d
G1 phase
S phase
G2 phase
Fold Change expression Ring1b-/- / Ring1b fl/fl (Log2 RPM)
vPRC1/ cPRC1/ PRC2 (n=2093)
vPRC1 specific (n=6222)
e
Fold Change expression in G2 Ring1b-/- / Ring1b fl/fl (Log2 RPM)
90.7%
34.7%
0.29%
0.1%
vPRC1/cPRC1/PRC2 (n=2093)
vPRC1 specific (n=6222)
Expression in Ring1b fl/fl in G2 (Log2 RPM)

Supplementary Figure 5. Depletion of Ring1b barely perturbs the transcriptional repression of target genes in G1 phase.

(a) Average normalized reads of 3D chromatin interactions (number of interactions = 261) measured by Capture-C of control promoters that are not bound by RING1B+CBX7 involving at least one promoter (number of promoters=41) in *Ring1b*$^{fl/fl}$ (black line) and *Ring1b*$^{-/-}$ (red line) cells during G1 (left) and G2 (right) phases. X axis represents the distance from the summit of the interaction peak measured as a function of DpnII restriction fragments. Dashed lines show enrichment at distance-matched control sites from each promoter and interaction in the opposite direction.

(b) Boxplot of nascent RNA reads mapped to the proximal promoter region (TSS to +3Kb) of vPRC1/cPRC1/PRC2 promoters (top panel) and vPRC1 specific targets (bottom panel) in *Ring1b*$^{fl/fl}$ and *Ring1b*$^{-/-}$ cells in G1 (left), S (middle) and G2 (right) phases. Boxes show median and Q1-Q3 values. Whiskers denote the 1.5X the interquartile range. Mann-Whitney test was applied (*P<0.05, **P<0.01, ***P<0.001, ****P<0.0001).

(c) MA plot showing fold changes in nascent RNA between *Ring1b*$^{-/-}$ and *Ring1b*$^{fl/fl}$ cells at vPRC1/cPRC1/PRC2 (top panel) and vPRC1 specific (bottom panel) target genes during G1, S and G2 phases. Genes showing a fold change > 1.5 are represented as red dots. Number and percentage (relative to total group targets) of deregulated genes are indicated.

(d) Violin plots showing the fold change of nascent RNA mapped to the proximal promoter region (TSS to +3kb) in *Ring1b*$^{-/-}$ and *Ring1b*$^{fl/fl}$ cells at vPRC1/cPRC1/PRC2 targets (green) and vPRC1 specific (grey) target genes in G1, S and G2 phases. P values were calculated using Mann-Whitney test. Asterisks (****) mark statistically significant differences (P<0.0001).

(e) MA plot showing fold changes of nascent RNA mapped to the proximal promoter region (TSS to +3kb) between *Ring1b*$^{-/-}$ and *Ring1b*$^{fl/fl}$ cells at vPRC1/cPRC1/PRC2 (black dots) and vPRC1 specific (grey dots) target genes relative to gene expression in *Ring1b*$^{fl/fl}$ cells in G2 phase. Percentage of genes up or down-regulated (above threshold FC>1.5) are shown.

Source data are provided as a source data file.

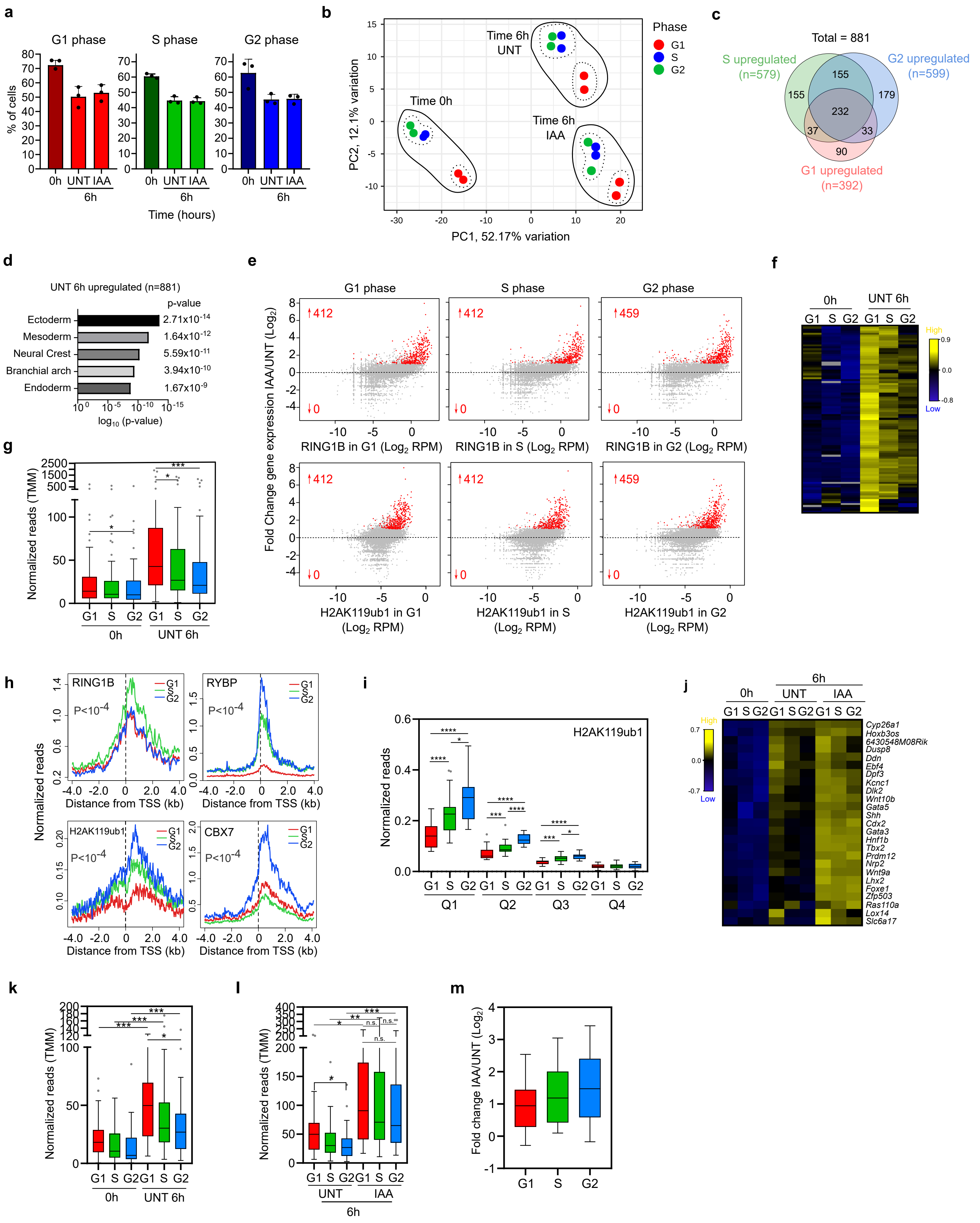
a
G1 phase
S phase
G2 phase
% of cells
0h UNT IAA
6h
Time (hours)
b
Time 6h UNT
Time 0h
Time 6h IAA
Phase
G1
S
G2
PC2, 12.1% variation
PC1, 52.17% variation
c
Total = 881
S upregulated (n=579)
G2 upregulated (n=599)
G1 upregulated (n=392)
155
155
179
232
37
33
90
d
UNT 6h upregulated (n=881)
p-value
Ectoderm 2.71x10-14
Mesoderm 1.64x10-12
Neural Crest 5.59x10-11
Branchial arch 3.94x10-10
Endoderm 1.67x10-9
log10 (p-value)
e
G1 phase
S phase
G2 phase
Fold Change gene expression IAA/UNT (Log2)
412
412
459
RING1B in G1 (Log2 RPM)
RING1B in S (Log2 RPM)
RING1B in G2 (Log2 RPM)
H2AK119ub1 in G1 (Log2 RPM)
H2AK119ub1 in S (Log2 RPM)
H2AK119ub1 in G2 (Log2 RPM)
f
0h
UNT 6h
G1 S G2 G1 S G2
High
Low
g
Normalized reads (TMM)
G1 S G2 G1 S G2
0h
UNT 6h
h
RING1B
RYBP
H2AK119ub1
CBX7
P<10-4
Normalized reads
Distance from TSS (kb)
i
H2AK119ub1
Normalized reads
G1 S G2 G1 S G2 G1 S G2 G1 S G2
Q1
Q2
Q3
Q4
j
6h
0h
UNT
IAA
G1 S G2 G1 S G2 G1 S G2
High
Low
Cyp26a1
Hoxb3os
6430548M08Rik
Dusp8
Ddn
Ebf4
Dpf3
Kcnc1
Dlk2
Wnt10b
Gata5
Shh
Cdx2
Gata3
Hnf1b
Tbx2
Prdm12
Nrp2
Wnt9a
Lhx2
Foxe1
Zfp503
Ras110a
Lox14
Slc6a17
k
Normalized reads (TMM)
G1 S G2 G1 S G2
0h
UNT 6h
l
Normalized reads (TMM)
G1 S G2 G1 S G2
UNT
IAA
6h
m
Fold change IAA/UNT (Log2)
G1
S
G2

Supplementary Figure 6. Treatment with retinoic acid induces the expression of ectoderm genes.

(a) Histogram showing the percentage of *Ring1a*$^{-/-}$;AID::*Ring1b*; in each phase of the cell cycle upon flow cytometry sorting in G1, S or G2 phases. Flow cytometry sorted cells (0h) were cultured in the presence (IAA) or absence (UNT) of IAA during six hours (6h) in differentiation media and fixed to be analysed by propidium iodide staining. Mean +/- SEM of three independent experiments is shown. No difference was found between UNT and IAA using Mann-Whitney test.

(b) Principal component analysis of gene expression of *Ring1a*$^{-/-}$;AID::*Ring1b*;FUCCI cells that had been cell cycle sorted (time 0h) and treated with RA for 6h. RING1B-depleted (time 6h, IAA) and control (time 6h UNT) cells are shown. Two biological replicates per experimental condition are represented.

(c) Venn diagram showing the overlap of 881 genes upregulated (FC>2, FDR<0.05) after 6h or RA treatment in cells sorted in the three different phases of interphase.

(d) Tissues gene ontology analysis of genes upregulated (FC>2, FDR<0.05, n=881) after 6h of RA treatment (identified in Supplementary Figure 6C). Fisher's exact test was used to calculate the P values.

(e) MA plot showing the fold changes in gene expression (TMM) between IAA-treated (IAA) and untreated (UNT) cells 6h upon RA stimulation. X-axis shows the enrichment of RING1B (top panel) or H2AK119ub1 (bottom panel) at the promoter (-0.5+1.5kb from TSS) of annotated genes in the mouse genome. Number of significantly up or down-regulated genes are indicated (FC>2, FDR<0.05).

(f) Hierarchical clustering of mRNA expression levels (TMM) of cell cycle sorted samples at 0h and 6h after RA treatment of genes preferentially upregulated during G1 compared to G2 phase (FC>2, n=97). Expression relative to the geometric mean of each gene is plotted.

(g) Boxplot of mRNA expression levels (TMM) of cell cycle sorted samples at 0h and 6h after RA treatment of genes preferentially upregulated during G1 compared to G2 phase (n=97).

(h) Average binding profile of RING1B, RYBP, H2AK119ub1 and CBX7 around the TSS of genes preferentially upregulated during G1 compared to G2 (n=97) in G1, S and G2 phases. *P* indicates one-way ANOVA test p-value.

(i) Boxplot showing H2AK119ub1 enrichment at the promoter region (-0.5 kb to +1.5 kb relative to TSS). Genes preferentially upregulated in G1 compared to G2 (described in Supplementary Figure 6F, n=97) were classified into quartiles based on H2AK119ub1 enrichment at G2 phase.

(j) Hierarchical clustering of mRNA expression (TMM) of cell cycle sorted samples at 0h and 6h after RA treatment in RING1B-depleted and control UNT cells. Genes preferentially upregulated during G1 compared to G2 phase that display higher levels of H2AK119ub1 (identified in Supplementary Figure 6I, n=25) are shown. Gene symbols are indicated. Expression relative to the geometric mean of each gene is plotted.

(k) Boxplot of mRNA expression (TMM) at 0h and 6h after RA treatment in cell cycle sorted cells. Genes preferentially upregulated during G1 compared to G2 phase that display higher levels of H2AK119ub1 (identified in Supplementary Figure 6I, n=25) are shown.

(l) Boxplot of mRNA expression (TMM) at 6h after RA treatment in cell cycle sorted RING1B-depleted and control UNT cells. Genes preferentially upregulated during G1 compared to G2 phase that display higher levels of H2AK119ub1 (identified in Supplementary Figure S6I, n=25) are shown.

(m) Boxplots showing the fold change of RNA expression (TMM) between IAA-treated and untreated cells at 6h after RA treatment of genes in Q1 (n=25) identified in Supplementary Figure 6I in cell cycle sorted cells.

(g, i, k, l, m) Boxes show median and Q1-Q3 values. Whiskers denote the 1.5X the interquartile range. Mann-Whitney test was applied (*P<0.05, **P<0.01, ***P<0.001, ****P<0.0001).

Source data are provided as a source data file.
