# Supplementary material for: Changes in PRC1 activity during interphase modulate lineage transition in pluripotent cells
Source: Nat Commun. 2023 Jan 12;14:180. doi: 10.1038/s41467-023-35859-9 (PMC9837203; doi:10.1038/s41467-023-35859-9)
Supplement: Supplementary file 2 — Description of Additional Supplementary Files [file 41467_2023_35859_MOESM2_ESM.pdf]

Description of Additional Supplementary Files

File Name: Supplementary Data 1
Description: Purity check of cell cycle–sorted FUCCI-mESCs used in this study.

File Name: Supplementary Data 2
Description: List of genes used in this manuscript.

File Name: Supplementary Data 3
Description: Raw data of colony forming assays.

File Name: Supplementary Data 4
Description: ChIP-seq enrichment and expression values.

File Name: Supplementary Data 5
Description: Reagents and published datasets used in this manuscript.
