# Supplementary material for: Changes in PRC1 activity during interphase modulate lineage transition in pluripotent cells
Source: Nat Commun. 2023 Jan 12;14:180. doi: 10.1038/s41467-023-35859-9 (PMC9837203; doi:10.1038/s41467-023-35859-9)
Supplement: Supplementary file 8 — Reporting Summary [file 41467_2023_35859_MOESM8_ESM.pdf]

nature portfolio

Corresponding author(s): David Landeira

Last updated by author(s): 02/12/2022

Reporting Summary

Nature Portfolio wishes to improve the reproducibility of the work that we publish. This form provides structure for consistency and transparency in reporting. For further information on Nature Portfolio policies, see our Editorial Policies and the Editorial Policy Checklist.

Statistics

For all statistical analyses, confirm that the following items are present in the figure legend, table legend, main text, or Methods section.

n/a	Confirmed	
☐	☒	The exact sample size (*n*) for each experimental group/condition, given as a discrete number and unit of measurement
☐	☒	A statement on whether measurements were taken from distinct samples or whether the same sample was measured repeatedly
☐	☒	The statistical test(s) used AND whether they are one- or two-sided<br>*Only common tests should be described solely by name; describe more complex techniques in the Methods section.*
☒	☐	A description of all covariates tested
☐	☒	A description of any assumptions or corrections, such as tests of normality and adjustment for multiple comparisons
☐	☒	A full description of the statistical parameters including central tendency (e.g. means) or other basic estimates (e.g. regression coefficient) AND variation (e.g. standard deviation) or associated estimates of uncertainty (e.g. confidence intervals)
☐	☒	For null hypothesis testing, the test statistic (e.g. *F*, *t*, *r*) with confidence intervals, effect sizes, degrees of freedom and *P* value noted<br>*Give P values as exact values whenever suitable.*
☒	☐	For Bayesian analysis, information on the choice of priors and Markov chain Monte Carlo settings
☒	☐	For hierarchical and complex designs, identification of the appropriate level for tests and full reporting of outcomes
☐	☒	Estimates of effect sizes (e.g. Cohen's *d*, Pearson's *r*), indicating how they were calculated

Our web collection on statistics for biologists contains articles on many of the points above.

Software and code

Policy information about availability of computer code

Data collection	Flow cytometry' module: Aria Fusion 1.0
Data analysis	STAR 2.5.2 , SAMtools 1.3.1 , MACS2, CoverageView 1.20.0, GraphPad 7, Enrichr 1.0, HiCUP Bowtie 2, CHiCAGO 1.0, deepTools 2, DESeq2

For manuscripts utilizing custom algorithms or software that are central to the research but not yet described in published literature, software must be made available to editors and reviewers. We strongly encourage code deposition in a community repository (e.g. GitHub). See the Nature Portfolio guidelines for submitting code & software for further information.

Data

Policy information about availability of data

All manuscripts must include a data availability statement. This statement should provide the following information, where applicable:

- Accession codes, unique identifiers, or web links for publicly available datasets
- A description of any restrictions on data availability
- For clinical datasets or third party data, please ensure that the statement adheres to our policy

Datasets are available at GEO-NCBI with accession number GSE207997 with a private token qvajggcyrpwzpoh that will be released upon publication acceptance.

Human research participants

Policy information about studies involving human research participants and Sex and Gender in Research.

Reporting on sex and gender	none
Population characteristics	none
Recruitment	none
Ethics oversight	none

Note that full information on the approval of the study protocol must also be provided in the manuscript.

Field-specific reporting

Please select the one below that is the best fit for your research. If you are not sure, read the appropriate sections before making your selection.

- [x] Life sciences
- [ ] Behavioural & social sciences
- [ ] Ecological, evolutionary & environmental sciences

For a reference copy of the document with all sections, see nature.com/documents/nr-reporting-summary-flat.pdf

Life sciences study design

All studies must disclose on these points even when the disclosure is negative.

Sample size	Sample size were the set of genes with given features. We did not perform sample size calculation.
Data exclusions	no data exclusion.
Replication	Independent biological triplicates were routinely carried out.
Randomization	Not relevant to this study.
Blinding	Not applicable.

Reporting for specific materials, systems and methods

We require information from authors about some types of materials, experimental systems and methods used in many studies. Here, indicate whether each material, system or method listed is relevant to your study. If you are not sure if a list item applies to your research, read the appropriate section before selecting a response.

Materials & experimental systems

n/a	Involved in the study	
	x	Antibodies
	x	Eukaryotic cell lines
x		Palaeontology and archaeology
x		Animals and other organisms
x		Clinical data
x		Dual use research of concern

Methods

n/a	Involved in the study	
	x	ChIP-seq
	x	Flow cytometry
x		MRI-based neuroimaging

Antibodies

Antibodies used

Ring1b Cell signalling D22F2
Ring1b MBL D139-3
Cbx7 Abcam ab21873
Rybp Millipore AB3637
H2Ak119ub1 Cell signalling 8240
Anti-mouse IgG Dako Z0259
LaminB Santa Cruz Sc-6216
Total H3 Abcam ab1791

Validation	All used antibodies are Western and ChIP grade and validated by manufacturer.

Eukaryotic cell lines

Policy information about cell lines and Sex and Gender in Research

Cell line source(s)	Mouse ESCs derived from mouse blastocysts and geneticly modified in vitro. Wild-type FUCCI-mESCs, Ring1a–/–;Ring1bfl/fl;Rosa26::CreERT2-FUCCI, AID::Ring1b;FUCCI
Authentication	Flow cytometry sorting of FUCCI cells, pluripotency markers analysis, western blot of target proteins and chip-pcr/seqs for Ring1b mutants.
Mycoplasma contamination	Routinely tested: negative
Commonly misidentified lines (See ICLAC register)	none

ChIP-seq

Data deposition

☒ Confirm that both raw and final processed data have been deposited in a public database such as GEO.

☒ Confirm that you have deposited or provided access to graph files (e.g. BED files) for the called peaks.

Data access links
May remain private before publication.

https://www.ncbi.nlm.nih.gov/geo/query/acc.cgi?acc=GSE207997

Files in database submission

GSM6329684 Ring1b-G1
GSM6329685 Ring1b-S
GSM6329686 Ring1b-G2
GSM6329687 Ring1b-MBL-G1
GSM6329688 Ring1b-MBL-S
GSM6329689 Ring1b-MBL-G2
GSM6329690 Ring1b-MBL-WT
GSM6329691 Ring1b-MBL-KO
GSM6329692 Rybp-G1
GSM6329693 Rybp-S
GSM6329694 Rybp-G2
GSM6329695 H2AK119ub1-G1
GSM6329696 H2AK119ub1-S
GSM6329697 H2AK119ub1-G2
GSM6329698 Cbx7-G1
GSM6329699 Cbx7-S
GSM6329700 Cbx7-G2
GSM6329701 RNA-Ring1bflfl-G1
GSM6329702 RNA-Ring1bflfl-S
GSM6329703 RNA-Ring1bflfl-G2
GSM6329704 RNA-Ring1bKO-G1
GSM6329705 RNA-Ring1bKO-S
GSM6329706 RNA-Ring1bKO-G2
GSM6329707 RNA-0h-G1-R1
GSM6329708 RNA-0h-G1-R2
GSM6329709 RNA-0h-S-R1
GSM6329710 RNA-0h-S-R2
GSM6329711 RNA-0h-G2-R1
GSM6329712 RNA-0h-G2-R2
GSM6329713 RNA-6h-UNT-G1-R1
GSM6329714 RNA-6h-UNT-G1-R2
GSM6329715 RNA-6h-UNT-S-R1
GSM6329716 RNA-6h-UNT-S-R2
GSM6329717 RNA-6h-UNT-G2-R1
GSM6329718 RNA-6h-UNT-G2-R2
GSM6329719 RNA-6h-IAA-G1-R1
GSM6329720 RNA-6h-IAA-G1-R2
GSM6329721 RNA-6h-IAA-S-R1
GSM6329722 RNA-6h-IAA-S-R2
GSM6329723 RNA-6h-IAA-G2-R1
GSM6329724 RNA-6h-IAA-G2-R2
GSM6329725 Ring1bflfl-G1-Six2-prom

	GSM6329726 Ring1bflfl-G2-Six2-prom<br>GSM6631348 Ring1b-G1-Input<br>GSM6631349 Ring1b-S-Input<br>GSM6631350 Ring1b-G2-Input<br>GSM6631351 Ring1b-MBL-WT-Input<br>GSM6631352 Ring1b-MBL-KO-Input<br>GSM6631353 Rybp-H2AK119ub1-Cbx7-Input<br>GSM6631354 Ring1bflfl-G1<br>GSM6631355 Ring1bflfl-G2<br>GSM6631356 Ring1bKO-G1<br>GSM6631357 Ring1bKO-G2
Genome browser session (e.g. UCSC)	https://genome.ucsc.edu/s/jordi.martorell/PRC1

Methodology

Replicates	Chips were analyzed by chip-pcr triplicates using published primers for positive and negative control regions. Libraries of representative samples were synthesized and sequenced.
Sequencing depth	20-30 million reads [50–base pair (bp) single reads] were obtained for each library.
Antibodies	Cell signalling D22F2<br>MBL D139-3<br>Abcam ab21873<br>Millipore AB3637<br>Cell signalling 8240<br>Dako Z0259<br>Santa Cruz Sc-6216
Peak calling parameters	STAR --readFilesIn sample.fastq.gz --genomeDir ./STAR_genome_mm9/ --runThreadN 16 --outFileNamePrefix star_mapping/sample --outSAMtype BAM SortedByCoordinate --outWigType wiggle --outWigNorm RPM --outWigStrand Unstranded<br><br>macs2 callpeak -c star_mapping/INPUT.bam -t star_mapping/sample.bam -f BAM -g mm -n peaks/sample.bam -B --SPMR -p 0.001
Data quality	Comparison of peak number, peaks profiles (by IGV) and enrichment (through correlation analyses) with published data.<br>Ring1b (FDR 0.01): total peaks G1(16009), S (18129), G2(15108)<br>Ring1b-MBL (FDR 0.05): total peaks G1(4488), S (8748), G2(5590)<br>Rybp (FDR 0.01): total peaks G1(4233), S (17880), G2(15525)<br>H2AK119ub1 (p-value<0.001): total peaks G1(234), S (753), G2(1588)<br>Cbx7 (FDR 0.05): total peaks G1(3810), S (3473), G2(5219)
Software	STAR 2.5.2 , SAMtools 1.3.1 , MACS2, CoverageView 1.20.0, GraphPad 7, Enrichr (www.maayanlab.cloud/Enrichr),

Flow Cytometry

Plots

Confirm that:

- [x] The axis labels state the marker and fluorochrome used (e.g. CD4-FITC).
- [x] The axis scales are clearly visible. Include numbers along axes only for bottom left plot of group (a 'group' is an analysis of identical markers).
- [x] All plots are contour plots with outliers or pseudocolor plots.
- [x] A numerical value for number of cells or percentage (with statistics) is provided.

Methodology

Sample preparation	Live cells were resuspended in buffer for sorting.
Instrument	Aria Fusion
Software	Aria Fusion software.
Cell population abundance	Please, see table S1.
Gating strategy	As shown in figure S1A, live cells were gated depending on their expression of Citrine and Cherry flurorescent proteins.

- [x] Tick this box to confirm that a figure exemplifying the gating strategy is provided in the Supplementary Information.
